# Evaluierung des GeSRU-Steps-Lehrvideokonzepts (German Society of Residents in Urology e. V.)

**DOI:** 10.1007/s00120-023-02248-5

**Published:** 2023-12-28

**Authors:** Tim Nestler, Emrah Hircin, Carolin Siech, Nadim Moharam, Angelika Mattigk, Hendrik Borgmann, Timur H. Kuru, Johannes Salem

**Affiliations:** 1 Klinik für Urologie, Bundeswehrzentralkrankenhaus Koblenz, Rübenacherstr. 170, 56072 Koblenz, Deutschland; 2 Klinik für Urologie, Uniklinik Köln, Köln, Deutschland; 3 AMBOSS GmbH, Köln, Deutschland; 4 Universitätsklinik, Klinik für Urologie, Goethe Universität Frankfurt, Frankfurt, Deutschland; 5 Cancer Prognostics and Health Outcomes Unit, Division of Urology, University of Montréal Health Center, Montréal, Québec, Kanada; 6 Klinik für Urologie, Universitätsklinikum Mannheim, Mannheim, Deutschland; 7 Klinik für Urologie und Kinderurologie, Universitätsklinikum Ulm, Ulm, Deutschland; 8 Klinik für Urologie und Kinderurologie, Universitätsklinikum Brandenburg a.d. Havel, MHB Brandenburg Theodor Fontane, Neuruppin, Deutschland; 9 CUROS urologisches Zentrum, Klinik Links vom Rhein, Köln, Deutschland

**Keywords:** Operationsvideo, Operationsausbildung, Strukturierte Ausbildung, Videoplattform, Assistenzarzt, Surgical video, Surgical training, Structured training, Video platform, Medical resident

## Abstract

**Hintergrund:**

Operative Lehrvideos stellen eine zeitgemäße, multimediale Ergänzung der operativen Aus- und Weiterbildung dar. Die German Society of Residents in Urology e. V. (GeSRU) entwickelte eine Lehrvideoplattform (steps.GeSRU.de) mit kostenfreien und qualitätsgesicherten Lehrvideos speziell für junge Ärzte in urologischer Weiterbildung.

**Ziel der Arbeit:**

Das Ziel der Arbeit war die Evaluierung des prinzipiellen Konzepts der GeSRU-Steps-Lehrvideos.

**Material und Methoden:**

Prospektiv wurden 29 GeSRU-Steps-Lehrvideos (03/2019–05/2023) über amboss.com bereitgestellt und ein Online-Fragebogen im Nachgang an die Videos eingeblendet. Dieser umfasste 12 Items zur medizinischen, technischen und didaktischen Qualität, zur Nützlichkeit für den eigenen Wissenserwerb und zu soziodemografischen Daten der Befragten. Aspekte der Videoqualität wurden mit der Acceptability-e-Scale sowie dem Global Quality Score untersucht.

**Ergebnisse:**

Im Erhebungszeitraum wurden die auf der Web-Seite amboss.com implementierten GeSRU-Steps-Videos 49.698-mal aufgerufen. Insgesamt wurden 474 Fragebögen beantwortet (Quote: 0,25 %). Das Kollektiv der Befragten bestand aus 419 (88 %) Studierenden, 47 (10 %) Ärzten in Weiterbildung und 5 (1 %) Fachärzten; 351 (74 %) waren weiblich, 107 (23 %) waren männlich und 4 (1 %) waren divers. Bewertet wurde jedes Lehrvideo im Median 10-mal (Range: 5‑ bis 65-mal). Die 6 Fragen der Acceptability-e-Scale und des Global Quality Scores wurden jeweils mit gut und sehr gut (81,6–95,8 %) bewertet.

**Diskussion:**

Die GeSRU-Lehrvideos sind ein zeitgemäßes Weiterbildungstool und erreichten eine sehr gute Bewertung mit hoher Zufriedenheit der Nutzer. Durch gezielte Förderung dieser qualitätsgesicherten Lehrmethode kann das Portfolio der niederschwellig verfügbaren Operationsvideos erweitert werden.

**Zusatzmaterial online:**

Die Online-Version dieses Beitrags (10.1007/s00120-023-02248-5) enthält eine zusätzliche Tabelle. Beitrag und Zusatzmaterial stehen Ihnen im elektronischen Volltextarchiv auf https://www.springermedizin.de/urologie zur Verfügung. Sie finden das Zusatzmaterial am Beitragsende unter „Supplementary Information“.

## Abstract (en)

**Background:**

Surgical educational videos represent a contemporary, multimedia supplement to surgical education and training. The German Society of Residents in Urology e. V. (GeSRU) developed an educational video platform (steps.GeSRU.de) with free, quality-assured educational videos for urologists, especially for residents.

**Objectives:**

The purpose of this study was to evaluate the GeSRU Steps teaching videos.

**Materials and methods:**

Prospectively, 29 GeSRU Steps training videos were made available (03/2019–05/2023) via amboss.com, and an online questionnaire was inserted following the videos. This comprised 12 items on medical, technical, and didactic quality, usefulness for own knowledge acquisition, and sociodemographic data of respondents. Aspects of video quality were assessed with the Acceptability E‑scale and the Global Quality Score.

**Results:**

During the survey period, the GeSRU Steps videos implemented on the amboss.com website were viewed 49,698 times. A total of 474 questionnaires were answered (rate 0.25%). The collective of respondents consisted of 419 (88%) students, 47 (10%) physicians in training, and 5 (1%) specialists; 351 (74%) were female, 107 (23%) were male, and 4 (1%) were diverse. Each educational video was rated a median of 10 times (range 5–65). The six questions of the Acceptability E‑scale and the Global Quality Score were rated good and very good (81.6–95.8%), respectively.

**Conclusions:**

GeSRU teaching videos achieved a very good rating with high user satisfaction. By specific promotion of these teaching videos, which are quality-assured through supervision, the portfolio of surgical videos available at a low threshold can be expanded and can serve as a contemporary education tool.

Die German Society of Residents in Urology e. V. (GeSRU) stellt eine kostenfreie Lehrvideoplattform mit Operationslehrvideos bereit (steps.gesru.de), um eine zeitgerechte Ausbildung zu ermöglichen. Das Ziel dieser Arbeit war die Evaluierung der einheitlichen Methodik, nach der die Lehrvideos erstellt werden.

Operative Fähigkeiten können auf unterschiedliche Weise vermittelt werden, per direktem Teaching im Operationssaal (OP), mittels OP-Lehrbuch oder als Lehrvideo. Durch immer weiterreichende Angebote in den neuen Medien verändert sich auch die Nachfrage nach zeitgemäßen Formaten. Wurden eingangs Lehrbücher digitalisiert und online verfügbar gemacht, gehören heute dank schneller Internetverbindungen Videos zum multimedialen Alltag [1].

Es existieren eine Vielzahl an unterschiedlichen Online-Angeboten, die urologische OP-Videos anbieten. Hier sind neben Angeboten der verschiedenen urologischen Fachgesellschaften auch Videoportale wie bspw. YouTube zu nennen. Diese stellen jedoch häufig seltene OP, OP für fortgeschrittene Operateure oder nur Ausschnitte aus einer Operation dar. Vermeintlich banale Eingriffe wie die Zirkumzision finden sich häufig jedoch nicht. Weiter sind die Videoportale der Fachgesellschaften in der Regel nur Mitgliedern passwortgeschützt zugänglich. Darüber hinaus gibt es meist keine Hinweise, wie die Qualität der Videos gesichert wird. Besonders für Kollegen zu Beginn ihrer beruflichen Tätigkeit und geringer Erfahrung ist somit eine Einordnung der unterschiedlichen Videoqualitäten schwierig bis unmöglich. Meist werden Videos unterschiedlichster Struktur und Aufbereitung ohne eine systematische Methodik dargeboten.

Operative Lehrvideos stellen somit eine zeitgemäße, multimediale Ergänzung der operativen Aus- und Weiterbildung von Ärzten dar. Der Bedarf an operativen Lehrvideos ergibt sich aus dem multimedialen Wandel unserer Gesellschaft [1]. Die Homepage der GeSRU wurde in 2016 grundlegend umgestaltet. Gleichzeitig wurde die GeSRU-Steps-Lehrvideoplattform integriert (steps.gesru.de). Ziel der GeSRU-Steps ist die Implementierung einer urologischen Videoplattform, die qualitativ hochwertige und qualitätsgesicherte Lehrvideos bereitstellt, um die operative Ausbildung urologischer Assistenzärzte zu ergänzen. Diese sollen die Vorbereitung auf den kommenden Eingriff erleichtern und zum kurzfristigen Auffrischen vor der entsprechenden Operation dienen.

Eine Auswahl der vorhandenen Lehrvideos (z. Z. 51 Videos) der Seite steps.gesru.de wurde prospektiv mittels Online-Befragung analysiert. Die Zufriedenheit der Nutzer und der Benefit für den klinischen Alltag wurden abgefragt. Das Ziel der Arbeit war die Evaluierung der GeSRU-Steps-Lehrvideos.

## Material und Methodik

### Konzept der GeSRU-Steps-Lehrvideos

Die Lehrvideos werden in einem einheitlichen Design und mit gleichem methodischem Aufbau gestaltet. Grundlage ist das „Step-by-step-Konzept“, bei dem eine Operation in verschiedene Abschnitte untergliedert wird, die der Reihe nach vorgestellt werden. Vor jedem Schritt wird eine kurze Erklärung gegeben und auf mögliche Schwierigkeiten oder Komplikationen hingewiesen. Zusätzlich besteht die Möglichkeit eines simultanen Audiokommentars zur detaillierteren Erläuterung des visuell Gezeigten. Mit einer Länge von 3 bis 5 min sollen die OP-Videos eine komprimierte Wissensauffrischung ermöglichen.

Die Sicherung der hohen fachlichen Qualität erfolgt durch Supervision des Chef- oder Oberarztes desjenigen Assistenten, der ein Video für einen der deutschen urologischen Kongresse erstellt und durch die Präsentation des Videos auf dem Kongress. Im Nachgang wird das Video dann der Videoplattform (steps.gesru.de) hinzufügt.

### Fragebogen

Der Online-Fragebogen zur Beurteilung und Bewertung des Lehrvideos umfasste insgesamt 12 Items zur medizinischen, technischen und didaktischen Qualität, zur Nützlichkeit für den eigenen Wissenserwerb, zur Weiterempfehlung und zu soziodemografische Daten der Befragten (Fragebogen: Zusatzmaterial Tab. 1). Aspekte der *Videoqualität* wurden mit 5 der 6 Fragen umfassenden Acceptability-e-Scale untersucht, die inhaltlich auf die hier zu untersuchenden Videos modifiziert wurden [2]. Diese waren mittels 5‑Punkte-Likert-Skala zu beantworten. Die Fragen umfassten die Aspekte der allgemeinen Zufriedenheit, der medizinischen Qualität, der technischen Qualität, der didaktischen Nützlichkeit und der zeitlichen Nützlichkeit. Weiter wurde die allgemeine Videoqualität mit dem ins Deutsche übersetzten Global Quality Score erfasst, ebenfalls mittels 5‑Punkte-Likert-Skala [3]. Wurde angegeben, dass der Zeitaufwand im Verhältnis zum vermittelten Inhalt unangemessen war, so wurde nachgefragt, ob das Video als zu lang oder zu kurz empfunden wurde. Der Abschnitt wurde mit der Freitextfrage beendet: „Was hat dir im Video gefehlt? Worauf hätte man deiner Meinung nach verzichten können?“.

Die erhobenen *soziodemografischen Daten* umfassten das Geschlecht (weiblich, männlich, divers, keine Angabe), den Ausbildungsstand (Student/in, Ärztin/Arzt in Weiterbildung, Fachärztin/Facharzt) und je nach Ausbildungsstand die Frage nach dem Semester, Jahr der ärztlichen Weiterbildung bzw. die Jahre als Facharzt. Abgeschlossen wurde die Befragung mit der offenen Frage: „Hast du noch weitere Anmerkungen? Wir freuen uns über dein Feedback!“.

### Verteilung des Fragebogens

Eine Auswahl von 29 GeSRU-Steps-Videos (Tab. 1) wurde im Jahr 2019 auf der Lehrplattform www.amboss.com in die entsprechenden Kapitel implementiert. Im Zeitraum von 25.03.2019 bis 08.05.2023 (49,5 Monate) wurde im Anschluss an diese Videos der oben genannten Fragebogen zur freiwilligen und anonymen Beantwortung eingeblendet. Über die Metadaten von amboss.com wurden weiterhin die Abrufzahlen der einzelnen Videos erfasst. Dieser Beitrag beinhaltet keine Studien an Menschen oder Tieren, weshalb kein Ethikvotum benötigt wurde.

**Tab. 1:** Auswahl von 29 GeSRU-Steps-Lehrvideos (German Society of Residents in Urology e. V.), die auf amboss.com implementiert wurden

Video	Aufrufe (*n* [%])
Ablatio testis	13 (2,7)
Ablatio testis – inguinal	9 (1,9)
Antegrade Varikozelensklerosierung nach Tauber	9 (1,9)
Bedside Surgery bei roboterassistierten Operationen – Tipps und Tricks Teil 1	8 (1,7)
Zirkumzision (1)	65 (13,7)
Zirkumzision (2)	20 (4,2)
DJ-Einlage mittels flexiblem Zytoskop (Ureterorenoskopie)	25 (5,3)
End-zu-End-Anastomose bei Harnröhrenstriktur	10 (2,1)
ESWL	7 (1,5)
Extraperitoneale laparoskopische Leistenherniotomie	8 (1,7)
Flexible URS (Steinbehandlung)	17 (3,6)
Hodentumorenukleation	8 (1,7)
Holmiumenukleation der Prostata (HoLEP)	7 (1,5)
Hydrozelenoperation	18 (3,8)
Hydrozelenresektion nach von Bergmann	8 (1,7)
Ileumneoblase	18 (3,8)
Laparoskopische Varikozelenligatur	8 (1,7)
Nephrektomie	22 (4,6)
Nephrostomie	22 (4,6)
Nierentumorenukleation	23 (4,9)
Retropubische Adenomenukleation der Prostata nach Millin	5 (1,1)
Sakrokolpopexie	6 (1,3)
Transurethrale Resektion in Saline (TURP/TURis, bipolar)	6 (1,3)
TUR-Blase	20 (4,2)
TUR-Prostata (1)	8 (1,7)
TUR-Prostata (2)	18 (3,8)
Ureterorenoskopie (1)	60 (12,7)
Ureterorenoskopie (2)	19 (4,0)
Uretherotomia interna nach Sachse	7 (1,5)
*Gesamt*	*474*

Die Häufigkeit der Videoaufrufe mit beantwortetem Fragebogen ist als absolute Zahl und relativ zur Gesamtkohorte *n* (%) angegeben

*ESWL* extrakorporale Stoßwellenlithotripsie, *TUR* transurethrale Resektion, *URS* Ureterorenoskopie

### Statistische Auswertung

Absolute und relative Häufigkeiten wurden für kategorische Variablen, Median und Range (Minimal- und Maximalwert) wurden für kontinuierliche Variablen angegeben. Der χ^2^-Test wurde für den Vergleich von nominal und ordinal skalierten Variablen verwendet, der t‑Test für intervallskalierte, unabhängige Stichproben. Alle statistischen Tests wurden 2‑seitig durchgeführt. Ein *p*-Wert < 0,05 wurde als signifikant angesehen. Die statistischen Berechnungen wurden mit IBM SPSS Statistics, Version 29 (Armonk, NY, USA) durchgeführt.

## Ergebnisse

In dem Erhebungszeitraum wurden die auf der Webseite amboss.com implementierten GeSRU-Steps-Videos 49.698-mal abgerufen. Insgesamt wurden 474 Online-Fragebögen im Anschluss an die GeSRU-Steps-Videos beantwortet (Tab. 1), entsprechend einer Quote von 0,25 % der Gesamtzahl der Videoaufrufe. Die Videos wurden im Mittel 16,3- sowie im Median 10-mal angesehen (Range: 5‑ bis 65-mal). In Tab. 1 sind die soziodemografischen Daten und die Ergebnisse der Befragung hinsichtlich der Videoqualität dargestellt.

### Soziodemografische Daten

An der Befragung nahmen 351 (74,1 %) Frauen, 107 (22,6 %) Männer und 4 (0,8 %) Diverse teil, 9 (1,9 %) gaben keine Antwort. Das Kollektiv teilte sich weiter in 419 (88,4 %) Studenten, 47 (9,9 %) Ärzte in Weiterbildung und 5 (1,1 %) Fachärzte auf. Die Studenten waren im Mittel im 10. Semester (Range: 1–16), die Ärzte in Weiterbildung im 2,6. Jahr (Range: 1–10) der Weiterbildung, die Fachärzte machten keine Angaben. Zwischen dem Geschlecht (weiblich/männlich) und dem Ausbildungsstand (Studenten/Ärzte in Weiterbildung) bestand kein signifikanter Unterschied.

### Videoqualität (Fragen 1–6)

Die Beantwortungsraten der Fragen zur Videoqualität waren: Fragen 1, 2, 5 und 6 jeweils *n* = 474 (100 %), Frage 3 *n* = 467 (98,5 %) und Frage 4 *n* = 471 (99,4 %). Die entsprechenden Antworten sind in Tab. 2 dargestellt. Mit gut (4/5) oder sehr gut (5/5) bewerteten 81,6 % der Befragten den ersten Eindruck des Videos, 91,5 % die Korrektheit und Verständlichkeit der Fakten, 84,3 % die technische Qualität des Videos und 86 % das Video als Hilfe, um den Lerninhalt gut und verständlich zu erfassen. Den Zeitaufwand zum Schauen des Videos bewerteten 95,8 % der Befragten als angemessen. Fast alle Befragten (93,5 %) bewerteten das jeweilige Video als hilfreich.

**Tab. 2:** Darstellung der Antworten zu den 6 Fragen mit Bezug zur Videoqualität

Frage	1 (sehr schlecht)	2	3	4	5 (sehr gut)	Kann ich nicht beurteilen
	*n* (%)					
1. *Wie ist dein erster Eindruck vom Video, wie zufrieden bist du damit?* Antworte einfach spontan aus dem Bauch heraus. (1) bedeutet, du bist sehr unzufrieden damit. (5) bedeutet, du bist sehr zufrieden. Mit den Zahlen dazwischen kannst du deine Meinung abstufen	24 (5,1)	24 (5,1)	39 (8,2)	173 (36,5)	214 (45,1)	0
2. Bitte gib an, inwieweit du folgenden Aussagen zustimmst. (1) bedeutet, du stimmst überhaupt nicht zu. (5) bedeutet, du stimmst voll und ganz zu. Mit den Zahlen dazwischen kannst du deine Meinung abstufen. *Das Video stellt medizinische Fakten korrekt und verständlich dar*	7 (1,9)	8 (2,2)	16 (4,4)	93 (25,4)	242 (66,1)	108
3. Bitte gib an, inwieweit du folgenden Aussagen zustimmst. (1) bedeutet, du stimmst überhaupt nicht zu. (5) bedeutet, du stimmst voll und ganz zu. Mit den Zahlen dazwischen kannst du deine Meinung abstufen. *Das Video hat eine gute technische Qualität*	10 (2,2)	17 (3,7)	38 (8,3)	119 (25,9)	268 (58,4)	15
4. Bitte gib an, inwieweit du folgenden Aussagen zustimmst. (1) bedeutet, du stimmst überhaupt nicht zu. (5) bedeutet, du stimmst voll und ganz zu. Mit den Zahlen dazwischen kannst du deine Meinung abstufen. *Das Video hilft mir den Lerninhalt gut und verständlich zu erfassen*	9 (2,0)	13 (2,9)	37 (8,2)	126 (28,0)	262 (58,2)	24
5. Wenn du an den im Video vermittelten Lerninhalt denkst: *Findest du den Zeitaufwand zum Schauen des Videos angemessen?*	0	1 (0,02)	18 (3,9)	82 (17,9)	357 (77,9)	16
6. *Wie hilfreich fandest du das Video (Qualität/Informationen)?*	4 (0,8)	1 (0,2)	35 (7,4)	305 (64,3)	129 (27,2)	–

Die Antworten „Kann ich nicht beurteilen“ wurden bei der relativen Betrachtung ausgeschlossen

Differenziert nach dem Geschlecht der Befragten (weiblich vs. männlich) ergab sich lediglich für Frage 1 (erster Eindruck des Videos) ein statistisch signifikanter Unterschied in der Bewertung zwischen beiden Gruppen (4,2 vs. 3,9/5; *p* = 0,024).

### Freitextfragen

Die erste Freitextfrage lautete „Was hat dir im Video gefehlt? Worauf hätte man deiner Meinung nach verzichten können?“. Diese wurde von 7 (1,4 %) Teilnehmern beantwortet. Neben vielen positiven Kommentaren wurden auch konstruktive Hinweise gegeben, wie der Wunsch nach einer genaueren Darstellung einzelner anatomischer Schichten oder eine bessere Markierung anatomischer Landmarken.

Die zweite Freitextfrage lautete „Hast du noch weitere Anmerkungen? Wir freuen uns über dein Feedback!“ und wurde von 64 (13,5 %) Teilenehmern beantwortet. Hier äußerten sich 25 Teilnehmer (39 %) zufrieden mit dem Video. 17 % (11 Teilnehmer) hatten inhaltliche Verbesserungsvorschläge. 25 Teilnehmer (39 %) fanden technische Aspekte verbesserungswürdig.

## Diskussion

Das Ziel der Arbeit war die Evaluierung der GeSRU-Steps-Lehrvideos und die Frage, ob die Methodik der durch Kongresse verifizierten Videos im Steps-Konzept zu qualitativen und nützlichen Videos führt.

Das Thema Lehrvideos in der Medizin wurde bereits in mehreren Studien untersucht. Nason et al. [4] berichteten über eine Verbesserung der Ausbildung beim männlichen Harnblasenkatheterismus durch ein entsprechendes YouTube-Video. Armstrong et al. [5] zeigten eine optimierte Psoriasisdiagnostik durch ein Lehrvideo auf einer entsprechenden dermatologischen Plattform.

Nach unserem Kenntnisstand findet sich im urologischen Bereich ein Defizit an qualitativ hochwertigen urologischen Operationsvideos für die ersten Abschnitte der Weiterbildung („Anfängeroperationen“). Aktuelle Untersuchungen aus dem Jahr 2021 von Eccles [6] unter urologischen Assistenzärzten zeigte zu 93 % YouTube als primäre Quelle für Lehrvideos. Die qualitätsgesicherte Videoplattform der American Urological Assosiation (AUA) war nur einer Minderheit bekannt. Neuere Erhebungen [7] aus dem Jahr 2023 zeigten faktisch dasselbe Bild mit einer > 80 %igen Nutzung von YouTube als Plattform für die Weiterbildung durch operative Lehrvideos. Das Problem einer fehlenden qualitätsgesicherten Plattform wurde im Jahr 2017 [8] durch eine Datenerhebung erkannt und dann durch den GeSRU und durch die GeSRU-Steps-Lehrvideoplattform ein Medium erschaffen, das diese Lücke schließen sollte. Die Operationsvideos sollten den Anspruch an eine hohe inhaltliche Qualität und eine gute technische Produktion haben, um urologischen Weiterbildungsassistenten eine weitere Möglichkeit zur Optimierung ihrer operativen Ausbildung zu bieten.

Das Konzept der Sicherung der hohen fachlichen Qualität musste praktikabel und zugleich sicher implementiert werden. Hierzu erfolge eine Supervision des Chefarztes desjenigen Assistenten, der ein Video erstellt und der Videoplattform hinzufügt. Die Lehrvideos wurden in einem einheitlichen Design und mit gleichem Aufbau gestaltet. Grundlage sollte für alle Operationen das „Schritt für Schritt Konzept“ sein.

Um mehr Assistenten in der Weiterbildung erreichen zu können wurden die zu diesem Zeitpunkt vorhandenen 29 Videos im Jahre 2019 in die Lernplattform amboss.com implementiert.

In der Summe wurden in allen Fragen hohe Werte vergeben (gut und sehr gut von 81,6–95,8 %, s. Tab. 2). Es sticht heraus, dass v. a. die Fragen nach dem zeitlichen Aufwand (Frage 5) für das Anschauen der Videos eine besonders gute Resonanz erfahren hat. Dies spiegelt in unseren Augen das veränderte Lernverhalten der jüngeren Kollegen dar. Das Lernen durch klassische Medien wie Bücher oder Atlanten wird zunehmend als zeitintensiv empfunden. Ein Video wird hier als der größere Ertrag in derselben Zeiteinheit wahrgenommen.

Der Hauptkritikpunkt der Teilnehmer lag in der technischen Umsetzung der Videos. Dies zeigt sich daran, dass mit Abstand die schlechtesten Werte in Frage 6 erreicht wurden (nur 27 % vergaben 5 Punkten). In der Auswertung der Freitextantworten wurde der technische Aspekt ebenfalls zu 39 % kritisiert. Dies lässt sich sicher damit erklären, dass die Lehrvideos von Ärzten erstellt wurden und nicht durch professionelle Ton- und Bildtechniker geschnitten und bearbeitet wurden. Aktuelle Studien zum Thema Weiterbildung aus dem Jahr 2023 von Li et al. [9] zeigten den Stellenwert eines einheitlichen nationalen Weiterbildungskonzept für angehende Assistenten auf. Über 70 % der befragten Assistenzärzte sprachen sich für ein abgestimmtes Konzept aus.

Einschränkend ist anzumerken, dass die hier vorgestellten Ergebnisse das Lehrvideoprojekt generell evaluieren. Ein Rückschluss auf einzelne Videos ist aufgrund der starken Schwankungsbreite der Antworten (5–65 Antworten pro Video) nicht möglich. Weiter wurde nur eine Auswahl der verfügbaren Lehrvideos evaluiert, da über die Zeit weitere Lehrvideos erstellt und in der Lehrvideoplattform implementiert wurden.

In Anbetracht der erhobenen Zufriedenheit sollten zunehmend Fördermittel in den Bereich Lehrvideos fließen, um die Ausbildung der angehenden Fachärzte weiter optimieren zu können. Zwischenzeitlich wurden die Lehrvideos über youtube.com noch einer weiteren Öffentlichkeit zugänglich gemacht.

## Schlussfolgerung

Die GeSRU-Lehrvideos mit dem Step-by-step-Konzept erreichten eine sehr gute Bewertung mit hoher Zufriedenheit der Nutzer. Die durch Supervision qualitätsgesicherte Lehrmethode kann die Ausbildung in der Urologie niederschwellig ergänzen und als zeitgemäßes Weiterbildungstool fungieren.

## Fazit für die Praxis

- Operationslehrvideos stellen eine zeitgemäße Methode zur Vorbereitung auf verschiedene Operationen dar.
- Urologische Operationslehrvideos, die sich inhaltlich „Anfängeroperationen“ verschrieben haben, finden sich qualitätsgesichert und kostenfrei auf steps.gesru.de.
- Der strukturierte Aufbau und die Kongressevaluation der Videos nach dem Step-by-step-Konzept wurden positiv evaluiert.
- Möchten Sie ein Operationslehrvideo erstellen und dieses gerne auf einem Kongress präsentieren, so wenden Sie sich an steps@gesru.de.

### Supplementary Information

Tab. 1 Fragebogen
