# Supplementary material for: Evaluation of the GeSRU-Steps educational video concept (German Society of Residents in Urology e. V.)
Source: Urologie. 2023 Dec 28;63(4):373–8. [Article in German] doi: 10.1007/s00120-023-02248-5 (PMC10991055; doi:10.1007/s00120-023-02248-5)
Supplement: Supplementary file 1 — Tab. 1 Fragebogen [file 120_2023_2248_MOESM1_ESM.pdf]

Zusatzmaterial

Tabelle 1: Fragebogen, wie er im Anschluss an die bei amboss.com eingebundenen GeSRU-Steps-Lehrvideos angezeigt wurde. Bei allen Fragen wurden Antwortmöglichkeiten vorgegeben, außer bei den Freitextfragen Nummer 8 und 14.

Nummer	Frage	Antworten						Anmerkung
1	Wie ist dein erster Eindruck vom Video, wie zufrieden bist du damit? Antworte einfach spontan aus dem Bauch heraus. (1) bedeutet, du bist sehr unzufrieden damit. (5) bedeutet, du bist sehr zufrieden. Mit den Zahlen dazwischen kannst du deine Meinung abstufen.	- (1) sehr unzufrieden	- (2)	- (3)	- (4)	- (5) sehr zufrieden		Allgemeine Zufriedenheit, modifiziert nach Acceptability E-scale [1]
2	Bitte gib an, inwieweit du folgenden Aussagen zustimmst. (1) bedeutet, du stimmst überhaupt nicht zu. (5) bedeutet, du stimmst voll und ganz zu. Mit den Zahlen dazwischen kannst du deine Meinung abstufen.Das Video...	... stellt medizinische Fakten korrekt & verständlich dar. - (1) stimme überhaupt nicht zu	... stellt medizinische Fakten korrekt & verständlich dar. - (2)	... stellt medizinische Fakten korrekt & verständlich dar. - (3)	... stellt medizinische Fakten korrekt & verständlich dar. - (4)	... stellt medizinische Fakten korrekt & verständlich dar. - (5) stimme voll und ganz zu	... stellt medizinische Fakten korrekt & verständlich dar. - Kann ich nicht beurteilen. 0	Medizinische Qualität, modifiziert nach Acceptability E-scale [1]
3	Bitte gib an, inwieweit du folgenden Aussagen zustimmst. (1) bedeutet, du stimmst überhaupt nicht zu. (5) bedeutet, du stimmst voll und ganz zu. Mit den Zahlen dazwischen kannst du deine Meinung abstufen.Das Video...	... hat eine gute technische Qualität (Bild, Ton etc.). - (1) stimme überhaupt nicht zu	... hat eine gute technische Qualität (Bild, Ton etc.). - (2)	... hat eine gute technische Qualität (Bild, Ton etc.). - (3)	... hat eine gute technische Qualität (Bild, Ton etc.). - (4)	... hat eine gute technische Qualität (Bild, Ton etc.). - (5) stimme voll und ganz zu	... hat eine gute technische Qualität (Bild, Ton etc.). - Kann ich nicht beurteilen. 0	Technische Qualität, modifiziert nach Acceptability E-scale [1]

4	Bitte gib an, inwieweit du folgenden Aussagen zustimmst. (1) bedeutet, du stimmst überhaupt nicht zu. (5) bedeutet, du stimmst voll und ganz zu. Mit den Zahlen dazwischen kannst du deine Meinung abstufen.Das Video...	... hilft mir, den Lerninhalt gut zu erfassen. - (1) stimme überhaupt nicht zu	... hilft mir, den Lerninhalt gut zu erfassen. - (2)	... hilft mir, den Lerninhalt gut zu erfassen. - (3)	... hilft mir, den Lerninhalt gut zu erfassen. - (4)	... hilft mir, den Lerninhalt gut zu erfassen. - (5) stimme voll und ganz zu	... hilft mir, den Lerninhalt gut zu erfassen. - Kann ich nicht beurteilen. (0)	Didaktische Nützlichkeit, modifiziert nach Acceptability E-scale [1]
5	Wenn du an den im Video vermittelten Lerninhalt denkst: Findest du den Zeitaufwand zum Schauen des Videos angemessen?	- unangemessen 1	- eher unangemessen 2	- teils, teils 3	- eher angemessen 4	- angemessen 5	- Kann ich nicht beurteilen. 0	Zeitliche Nützlichkeit, modifiziert nach Acceptability E-scale [1]
6	Wie hilfreich fandest du das Video?	1 - Generell nicht hilfreich: schlechte Qualität	2 - Sehr eingeschränkt hilfreich: schlechte Qualität, enthält jedoch wenige Informationen	3 - Teilweise hilfreich: mäßiger Fluss, einige Infos enthalten, aber wichtige Informationen fehlen	4 - Hilfreich: Gute Qualität und guter Fluss. Wesentliche Aspekte berücksichtigt	5 - Ausgesprochen hilfreich:exzellente(r) Qualität / Fluss		Modifizierter Global Quality Score [2]
7	Du hast angegeben, dass du den Zeitaufwand im Verhältnis zum vermittelten Inhalt (teilweise / eher) unangemessen findest. Findest du das Video zu lang oder zu kurz?	1 = zu kurz	2 = zu lang					
8	Was hat dir im Video gefehlt? Worauf hätte man deiner Meinung nach verzichten können?	offene Frage						
9	Geschlecht	1 = weiblich, 2 = männlich	2 = männlich	3 = divers	4 = keine Angabe			Soziodemographie
10	Du bist...	1 = Student/in	2 = Ärztin/Arzt in Weiterbildung	3 = Fachärztin/Facharzt				
11	In welchem Semester bist du?	1	2	3	4	5	6	
12	In welchem Jahr deiner ärztlichen Weiterbildung bist du?	1	2	3	4	5	6	

13	Seit wie vielen Jahren bist du bereits Facharzt/Fachärztin?	1	2	3	4	5	6	
14	Hast du noch weitere Anmerkungen? Wir freuen uns über dein Feedback!	offene Frage						

Literatur

1. Tariman, J.D.; Berry, D.L.; Halpenny, B.; Wolpin, S.; Schepp, K. Validation and Testing of the Acceptability E-Scale for Web-Based Patient-Reported Outcomes in Cancer Care. *Appl. Nurs. Res.* **2011**, *24*, 53–58, doi:10.1016/j.apnr.2009.04.003.

2. Garg, N.; Venkatraman, A.; Pandey, A.; Kumar, N. Online Information on Dialysis. *Nephrology* **2015**, *20*, 315–320, doi:10.1111/nep.12397.
